# Sleep duration, genetic susceptibility, and Alzheimer's disease: a longitudinal UK Biobank-based study

**DOI:** 10.1186/s12877-022-03298-8

**Published:** 2022-08-02

**Authors:** Shiqi Yuan, Wen Ma, Rui Yang, Fengshuo Xu, Didi Han, Tao Huang, MIn Peng, Anding Xu, Jun Lyu

**Affiliations:** 1 Department of Neurology, The First Affiliated Hospital of Jinan University, No.613, Huangpu Road West, Guangzhou, 510630 Guangdong Province China; 2 School of Public Health, Xi’an Jiaotong University Health Science Center, Xi’An, 710061 Shaanxi Province China; 3 Department of Clinical Research, The First Affiliated Hospital of Jinan University, No.613, Huangpu Road West, Guangzhou, 510630 Guangdong Province China

**Keywords:** Sleep duration, Genetic susceptibility, Alzheimer's disease (AD), Two Sample Mendelian randomization (MR), UK Biobank

## Abstract

**Background:**

Alzheimer's disease (AD) is the most frequently occurring type of dementia. Concurrently, inadequate sleep has been recognized as a public health epidemic. Notably, genetic and environmental factors are now considered contributors to AD progression.

**Objective:**

To assess the association between sleep duration, genetic susceptibility, and AD.

**Methods and results:**

Based on 483,507 participants from the UK Biobank (UKB) with an average follow-up of 11.3 years, there was a non-linear relationship between AD incidence and sleep duration (*P* for non-linear < 0.001) by restricted cubic splines (RCS). Sleep duration was categorized into short sleep duration (< 6 h/night), normal sleep duration (6–9 h/night), and long sleep duration (> 9 h/night). No statistically significant interaction was identified between sleep duration and the AD-GRS (Alzheimer's disease genetic risk score, *P* for interaction = 0.45) using Cox proportional risk model. Compared with the participants who had a low AD-GRS and normal sleep duration, there was associated with a higher risk of AD in participants with a low AD-GRS and long sleep duration (HR = 3.4806; 95% CI 2.0011–6.054, *p* < 0.001), participants with an intermediate AD-GRS and long sleep duration (HR = 2.0485; 95% CI 1.3491–3.1105, *p* < 0.001), participants with a high AD-GRS and normal sleep duration (HR = 1.9272; 95% CI 1.5361–2.4176, *p* < 0.001), and participants with a high AD-GRS and long sleep duration (HR = 5.4548; 95% CI 3.1367–9.4863, *p* < 0.001).In addition, there was no causal association between AD and sleep duration using Two Sample Mendelian randomization (MR).

**Conclusion:**

In the UKB population, though there was no causal association between AD and sleep duration analyzed using Two Sample MR, long sleep duration (> 9 h/night) was significantly associated with a higher risk of AD, regardless of high, intermediate or low AD-GRS. Prolonged sleep duration may be one of the clinical predictors of a higher risk of AD.

**Supplementary Information:**

The online version contains supplementary material available at 10.1186/s12877-022-03298-8.

## Background

Dementias are chronic progressive neurological diseases characterized by memory loss and cognitive impairment. Among the dementias, the most common is Alzheimer's disease (AD), which has a prevalence that is rising sharply as the global population ages [1, 2]. While AD is highly heritable [3], dementia research has focused on the potential impact of lifestyle factors, drug use, and health conditions on this disease [4].

Proper sleep is necessary for regulation of the body's metabolism. Studies have identified associations between sleep disorders and diabetes, obesity, and coronary heart disease, as well as other diseases [5]. In addition, inadequate sleep is considered a public health epidemic [6], where sleep can be influenced by many factors, including living and natural environments, mental variables, and diseases [7].

Studies showed there is a U-shaped or J-shaped curve between inappropriate sleep duration and AD risk [8, 9]. However, due to selection bias and relatively small numbers of participants, research findings were limited in previous studies. With the development of genomics and evolution of scientific research design, research in this area can be further improved. Moreover, genetic and environmental factors are now considered contributors to the progression of AD [10].

Observational studies cannot include all confounding factors and tend to result in reverse causality and thus are regarded as only limited causal inference [11]. Mendelian randomization (MR) based on genetic variations (i.e., single nucleotide polymorphisms, SNPs) can be used to further overcome these limitations [12]. MR calculates causal estimates of exposure risk using the random distribution of genetic variation during gametogenesis, independent of environmental confounding factors. Using this method, the degree of confounding can be significantly reduced and yield a low sensitivity to reverse causality [12, 13].

Therefore, we performed a longitudinal investigation on the association between sleep duration and AD risk utilizing the UK Biobank (UKB). In addition, we calculated the genetic risk score (GRS) for AD for each participant. The goal of this study was to assess the relationship between sleep duration and genetic susceptibility in terms of AD outcome and to further explore potential genetic/sleep interactions. Finally, we utilized Two Sample MR analysis to further characterize the causal relationship between sleep duration and AD.

## Methods

### Study population

Public database is a data repository specifically designed to house data related to scientific research on an open platform [14]. The UKB is the world's largest biomedical sample database designed to examine genetic and lifestyle risk factors for a variety of chronic diseases and houses data from a prospective population-based cohort study consisting of over half a million volunteers conducted from 2006 to 2010, The UK Biobank study was approved by the Northwest Multicenter Research Ethics Committee. All participants provided written informed consent  [15]. The UKB has collected and will continue to collect participant data on a large number of phenotypes and genotypes [16], including questionnaire-derived data, assayed samples, physical measurements, multimodal imaging, genome-wide genotyping, accelerometry, and longitudinal follow-up for a broad spectrum of health-related outcomes [15, 17].

Inclusion criteria: all UKB participants.

Exclusion criteria: participants with a diagnosis of AD; participants without complete sleep duration and genetic data; participants with an implausible sleep duration.

Finally, 483,507 participants were included in this study.

Increased age and vascular risk factors, including diabetes, hypertension, alcohol, smoking, Myocardial infarction, and stroke, are known to increase the risk of AD [18, 19]. Low socioeconomic status is associated with cognitive decline [18], low education level and ethnicity are the risk factor for AD [20, 21]. In addition, woman is also associated with higher AD risk [22]. Therefore, we selected the above variables as our baseline characteristics and confounding factors. The basic characteristics of each participant were determined primarily through registration or diagnostic records. All participants had complete case analysis.

### Ascertainment of exposure

Duration of sleep was self-reported by study participants based on the standardized question: “About how many hours sleep do you get in every 24 h? (Please include naps),” where responses were given in hours (https://bbams.ndph.ox.ac.uk). Individuals were excluded if they failed to provide their sleep duration or had sleep durations < 4 or > 18 h to reduce implausible sleep durations. Totally 4,394 participants were excluded. We applied the classification of sleep duration proposed by the National Sleep Foundation [23, 24], sleep duration was categorized into short sleep duration (< 6 h/night), normal sleep duration (6–9 h/night), and long sleep duration (> 9 h/night).

### Definition of genetic risk score

Quality control, input procedures, and genotyping for the UKB have been previously described [25]. In this study, 29 independent single nucleotide polymorphisms (SNPs) previously demonstrated to have a significant association with AD based on genome-wide association studies (GWASs) were selected [10, 26, 27]. The selected SNPs are detailed in Supplementary Table S1. For each individual in the UKB, an AD-GRS was determined using a previously published weighted method [28, 29] with the equation: weighted GRS = (β1x SNP1 + β2x SNP2 + … + βn x SNPn) x (N/sum of the β coefficient). The effect size (β-coefficient) of each SNP was derived from the reported GWAS results [27]. Using this method, each participant was assigned as either at high (quintile 5), intermediate (2–4 quartile), or low (quintile 1) genetic risk for the outcomes of interest.

### Townsend deprivation index

The Townsend deprivation index (TDI) is a composite score based on 4 key variables: unemployment, overcrowded household, non–car ownership, and non–home ownership. The index has been validated and used in population studies in the UK, with higher scores indicating higher levels of deprivation [30].

### Outcome assessment

Based on the UKB, each participant’s outcomes were identified primarily using hospital admissions data (time of AD diagnosis) and death registry records. Follow-up occurred from the registration date to the date of AD diagnosis, death, or experimental follow-up end date (December 2020), whichever occurred first.

## Statistical analyses

Comparisons between the baseline characteristics of Control group (Not diagnosed with AD) and AD group (Diagnosed with AD) were made using the Chi-square (categorical variable) or Student's t test (metric variable). Continuous variables are represented as mean with standard deviation or median with quartile range. The potential non-linear association between sleep duration and AD was investigated using restricted cubic splines (RCS). The Kaplan–Meier survival curve was utilized to more intuitively display the risk of AD among the three sleep duration groups (short sleep duration, normal sleep duration, and long sleep duration).

The Cox proportional risk model was used to estimate the hazard ratio (HR) of AD, and sleep duration and genetic risk interaction for AD. Model 1. The objective of Model 1 was to evaluate the risk ratios of different sleep duration to AD events without adjusting for other confounding factors; Model 2. Increased age and woman were significantly associated with higher AD risk, and the objective of model 2 was to evaluate whether different sleep duration was associated with higher AD risk independently of age and gender; Model 3. Low socioeconomic status was associated with cognitive decline, low education level and ethnicity are the risk factor for AD, and the objective of Model 3 was to evaluate whether different sleep duration was associated with higher risk of AD independently of demographic, educational, and economic status factors (age, gender, ethnicity, TDI, and education level); Model 4. Vascular risk factors, including diabetes, hypertension, alcohol, smoking, Myocardial infarction, and stroke, were known to increase the risk of AD. The objective of Model 4 was to evaluate whether different sleep duration was associated with a higher risk of AD independently of demographic, educational, economic status, and cardiovascular risk factors.

Two Sample MR analyses: MR is a novel statistical approach that assesses the random combination of genes from parent to offspring during gamete formation and conception and provides a method of determining whether there is a causal nature for certain environmental exposures [12, 31]. In this method, genetic variables are considered instrumental variables (IVs) and utilized to make causal inferences from data [32]. In this study, MR analysis involved a two-sample design to allow the exposures and results to be measured in a non-overlapping data set, further reducing the false positive rate [33]. Beta-weighted sleep duration genetic instruments were set as the exposures (https://gwas.mrcieu.ac.uk/; GWAS ID: ukb-b-4424) with participant overlap with the UKB, as well as outcome data from a GWAS (https://gwas.mrcieu.ac.uk/; GWAS ID: ieu-b-2) lacking participant overlap with the UKB. Detailed information on the SNPs is presented in Supplementary Tables S1 and S2. Individuals participating in the GWAS were of predominantly European ancestry. the Two Sample MR package was used for MR analyses.

All statistical analyses were performed with the R package (version 4.1.0), where a *P* value < 0.05 was considered statistically significant.

## Results

### Basic characteristics of control and AD groups

A study sample flow diagram is presented in Supplementary material Figure S1. Initially, there were 502,490 participants enrolled. Once participants with a diagnosis of AD had been excluded (*n* = 18), 502,472 participants remained. Finally, participants with complete sleep duration (4–17 h/night) and genetic data (*n* = 483,507) were selected for this study. The mean follow-up time was 11.3 years.

We compared the basic characteristics of participants who developed AD (AD group, *n* = 919) and those who did not (Control group, *n* = 482,588). As shown in Table 1, the AD group had a higher age index and higher TDI (*p* < 0.05). Those that were male, had a history of smoking, or were of mixed race had higher rates of AD (*p* < 0.05), as did those with hypertension, diabetes, or a history of myocardial infarction or stroke (*p* < 0.05). Moreover, a lower education level and less sleep significantly correlated with the incidence of AD (*p* < 0.05).

**Table 1:** Comparison of the basic characteristics between the control and Alzheimer’s disease (AD) group

Characteristics	Control group	AD group	*P* value
Age (Median, IQR)	58 (50,63)	66 (62,68)	< 0.001
TDI (Median, IQR)	-2.2 (-3.7,0.5)	-2 (-3.6,1.1)	0.037
Gender (n, %)			< 0.001
Female	261,396 (54.2)	443 (48.2)	
Male	221,192 (45.8)	476 (51.8)	
Ethnicity (n, %)			0.003
White people	437,884 (91)	849 (92.4)	
Mixed people	17,807 (3.7)	43 (4.7)	
Other people	25,270 (5.3)	27 (2.9)	
Education (n, %)			< 0.001
College/University	157,154 (32.9)	172 (19.3)	
Other	320,081 (67.1)	718 (80.7)	
Smoking (n, %)			< 0.001
Never	263,316 (54.8)	431 (47.4)	
Previous	167,034 (34.7)	388 (42.7)	
Current	50,485 (10.5)	90 (9.9)	
Alcohol (n, %)			< 0.001
Never	20,987 (4.4)	73 (8)	
Previous	17,105 (3.5)	74 (8.1)	
Current	443,980 (92.1)	768 (83.9)	
Myocardial infarction (n, %)			
No	462,906 (95.9)	803 (87.4)	< 0.001
Yes	19,682 (4.1)	116 (12.6)	
Stroke (n, %)			< 0.001
No	470,248 (97.4)	817 (88.9)	
Yes	12,340 (2.6)	102 (11.1)	
Diabetes (n, %)			< 0.001
No	477,875 (99)	885 (96.3)	
Yes	4713 (1)	34 (3.7)	
Hypertension (n, %)			< 0.001
No	334,628 (69.3)	393 (42.8)	
Yes	147,960 (30.7)	526 (57.2)	
Sleep duration (Median, IQR)	7 (7,8)	7 (7,8)	< 0.001

### Sleep duration association with risk of AD

**Fig. 1:**
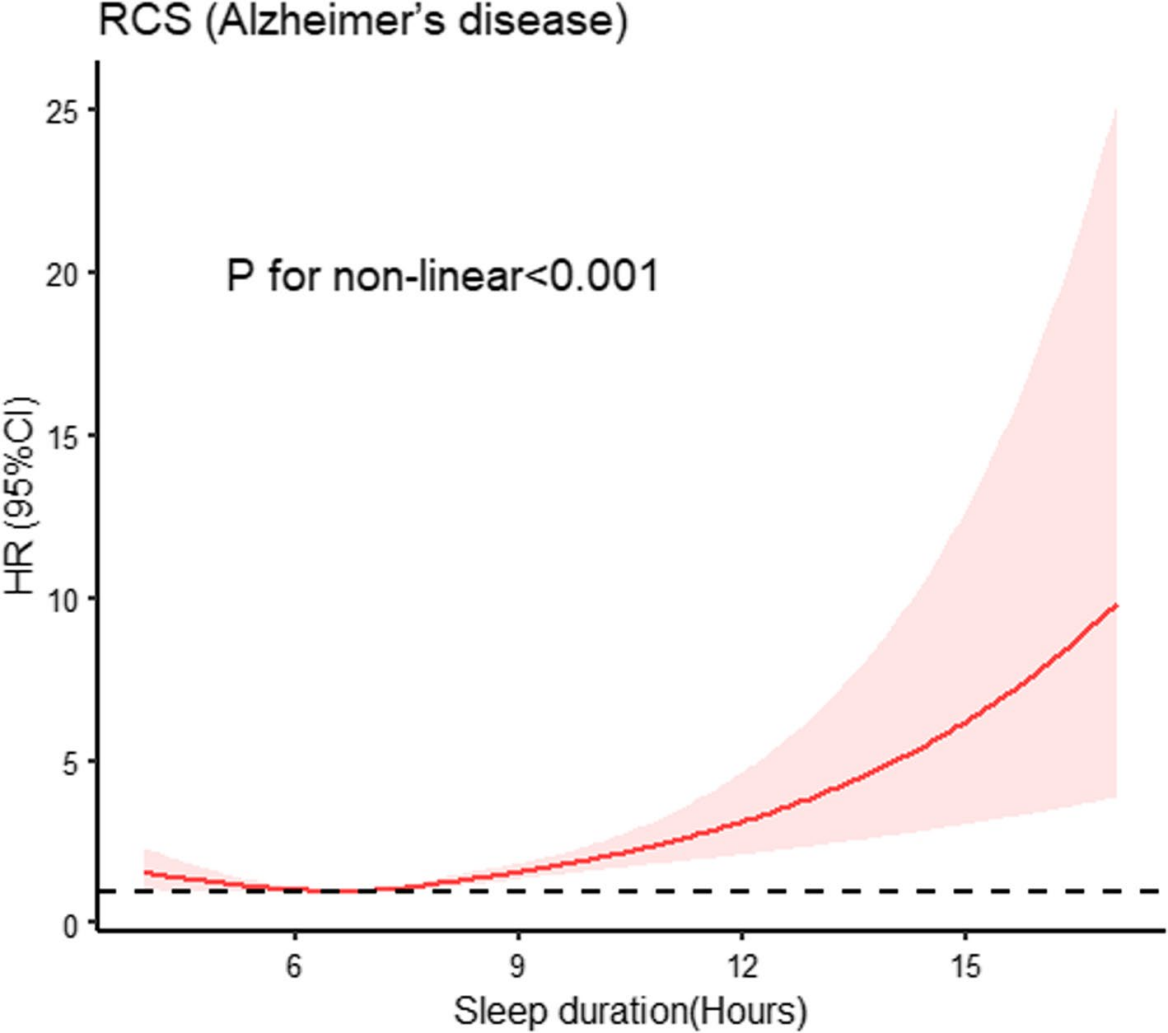
Restricted cubic splines (RCS) for analysis of the relationship between sleep duration and incidence of Alzheimer’s disease (AD). The model was adjusted for age, gender, ethnicity, TDI, education level, smoking, alcohol use, hypertension, stroke, myocardial infarction, and diabetes

The potential nonlinear association between AD and sleep duration was investigated using RCS. As shown in Fig. 1, there was a non-linear relationship between sleep duration and AD incidence (*P* for non-linear < 0.001). To further assess the association between different sleep durations and risk of AD, sleep duration was categorized into short sleep duration (< 6 h/night), normal sleep duration (6–9 h/night), and long sleep duration (> 9 h/night). The baseline data were grouped according to sleep duration, as shown in Supplementary material Table S3.

**Fig. 2:**
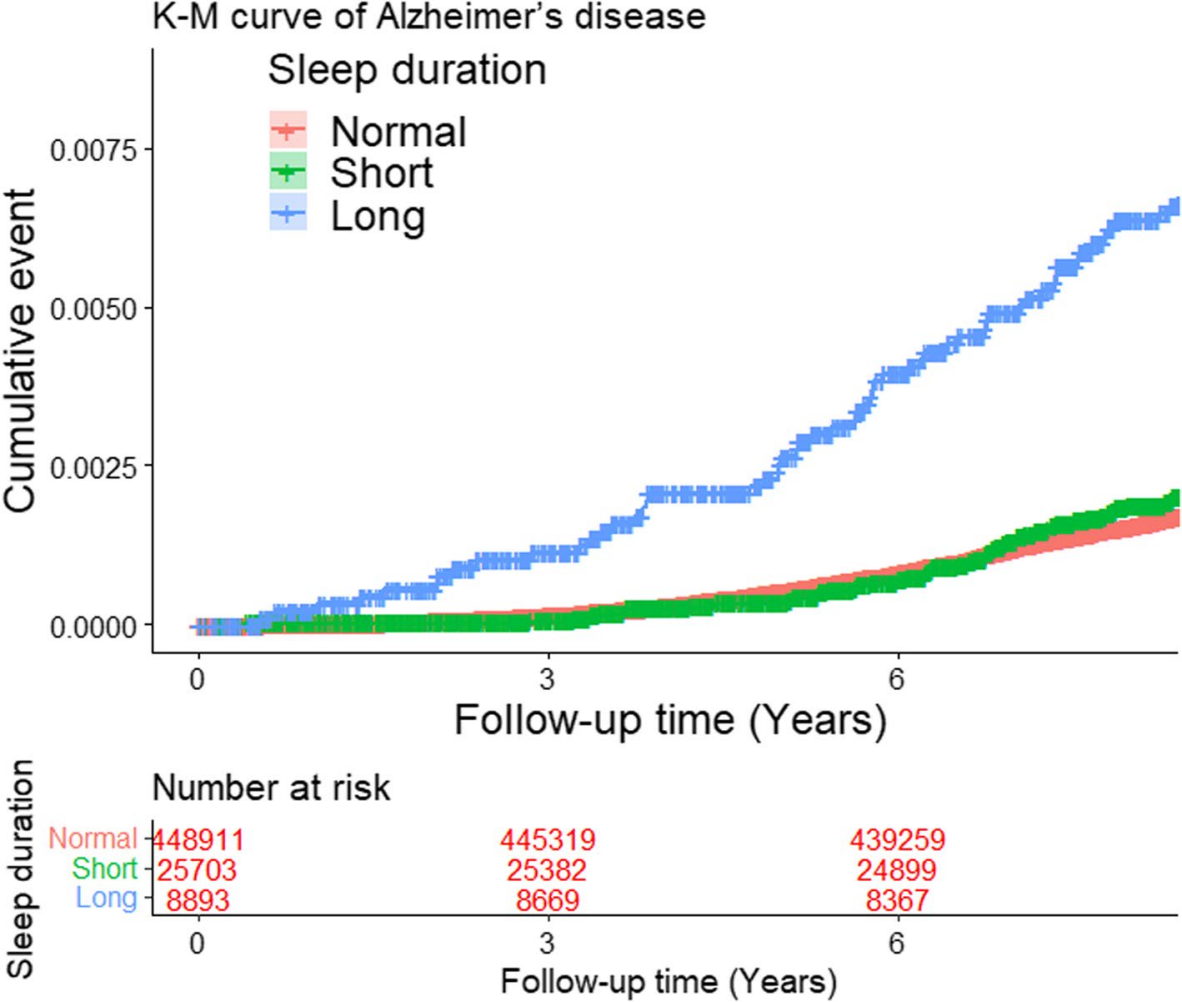
Kaplan–Meier survival curve demonstrating the risk of Alzheimer’s disease (AD) among the three sleep duration groups. The normal sleep duration group was used as the control group, and the difference between the two groups was evaluated by log-rank tests

Next, the Kaplan–Meier survival curve was utilized to more intuitively display the risk of AD among the three sleep duration groups. Concurrently, log-rank tests were used to evaluate whether there were statistically significant differences between groups. As shown in Fig. 2, the risk of AD in the long sleep group (*p* < 0.001) was higher than in the normal sleep duration group, while there was no significant difference between the normal and short sleep duration groups (*p* = 0.24).

### Hazard ratio estimation for AD

**Fig. 3:**
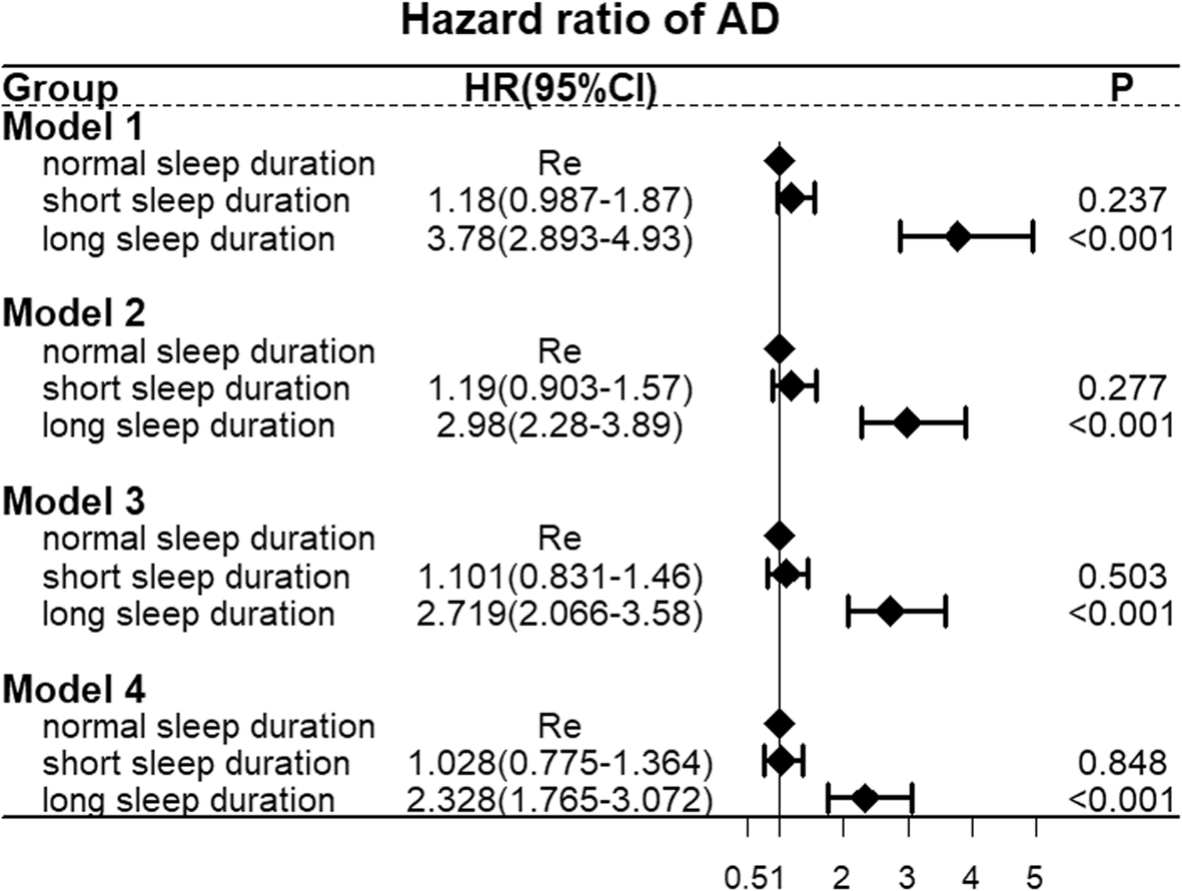
Cox proportional risk model estimating the hazard ratio of AD. Model 1 unadjusted; Model 2 adjusted for age and gender; Model 3 adjusted for terms in Model 2, ethnicity, TDI, and education level; Model 4 adjusted for terms in Model 3 and cardiovascular risk factors including smoking, alcohol use, hypertension, stroke, myocardial infarction, and diabetes. The vertical line indicates the reference value of 1

The Cox proportional risk model was used to estimate the HR of AD. As shown in Fig. 3, Compared with normal sleep duration group, long sleep duration group was associated with a higher risk of AD in unadjusted Model 1 (HR = 3.78, 95% CI 2.893–4.93, *P* < 0.001). This association was still significant after adjusting for age, gender, ethnicity, TDI, education level, smoking, alcohol use, hypertension, stroke, myocardial infarction, and diabetes in Model 4 (HR = 2.328, 95% CI 1.765–3.072, *P* < 0.001). In addition, after adjusting for multiple variables in Model 4, Compared with normal sleep duration group, there was still no significant association between short sleep duration group and the risk of AD (*P* > 0.05).

Our results demonstrated that the risk of AD in the long sleep group was still higher than in the normal sleep duration group after adjusting for multiple variables.

### Joint association between long sleep duration and AD genetic risk score

**Fig. 4:**
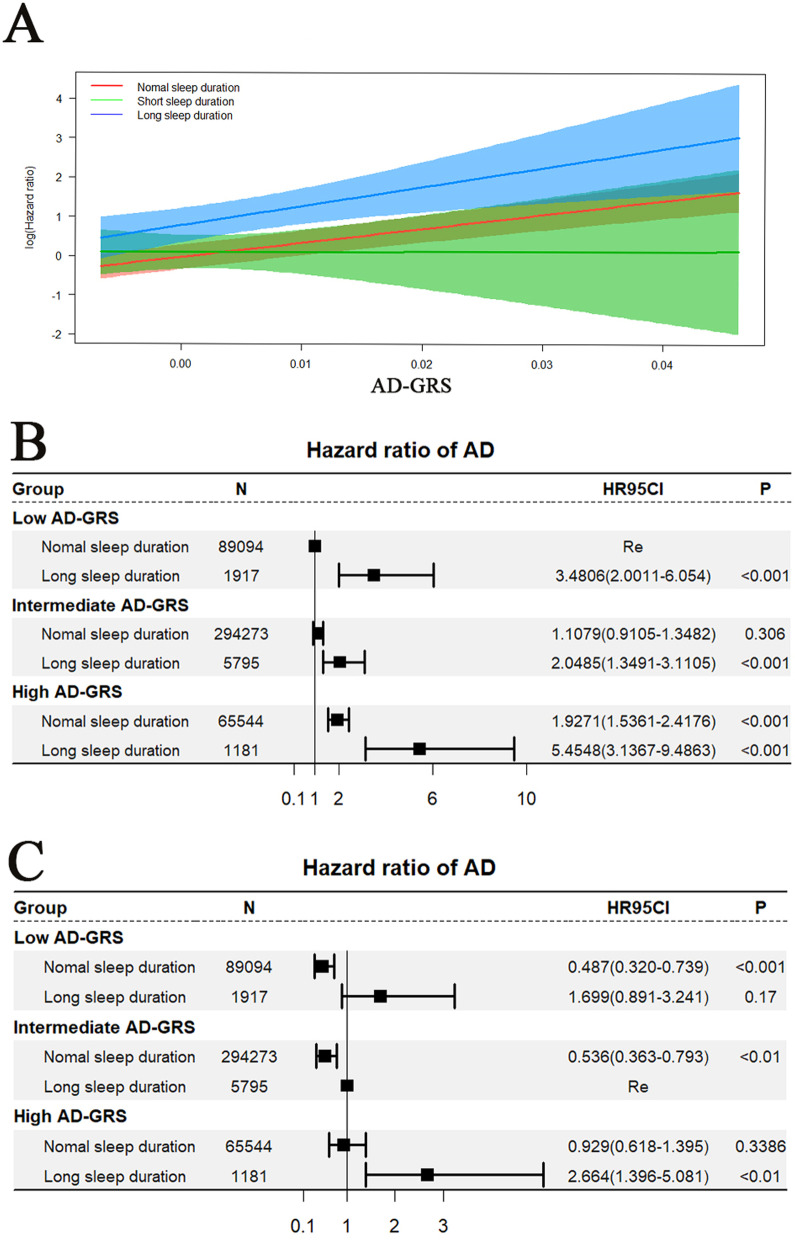
The joint association between long sleep duration and AD-GRS for AD risk. A: The interaction between sleep duration and AD-GRS. B: Participants who had a low AD-GRS with normal sleep duration were used as the reference (Re). C: Participants who had an intermediate AD-GRS with long sleep duration were used as the reference (Re). The multivariable model was adjusted for age, gender, ethnicity, TDI, education level, smoking, alcohol use, hypertension, stroke, myocardial infarction, and diabetes. The vertical line indicates the reference value of 1. AD-GRS stands for Alzheimer's disease genetic risk score

We further evaluated the joint association between sleep duration and AD-GRS. As shown in Fig. 4, no statistically significant interaction was identified between sleep duration and the AD-GRS (*P* for interaction = 0.45), and the visualization of this interaction is shown in Fig. 4A. As shown in Fig. 4B, compared with the participants who had both a low GRS and normal sleep duration, there was associated with a higher risk of AD in participants with a low AD-GRS and long sleep duration (HR = 3.4806; 95% CI 2.0011–6.054, *p* < 0.001), participants with an intermediate AD-GRS and long sleep duration (HR = 2.0485; 95% CI 1.3491–3.1105, *p* < 0.001), participants with a high AD-GRS and normal sleep duration (HR = 1.9272; 95% CI 1.5361–2.4176, *p* < 0.001), and participants with a high AD-GRS and long sleep duration (HR = 5.4548; 95% CI 3.1367–9.4863, *p* < 0.001). Fig. 4C showed that the participants with a low AD-GRS and long sleep duration had no significantly association with higher risk of AD compared with the participants with intermediate AD-GRS and long sleep duration (HR = 0.929; 95% CI 0.618–1.395, *p* > 0.05).

Our results indicated that the risk of AD increased as the AD-GRS increased, and long sleep duration was significantly associated with a higher risk of AD, regardless of high, intermediate or low AD-GRS.

### Two Sample MR of sleep duration and AD

**Fig. 5:**
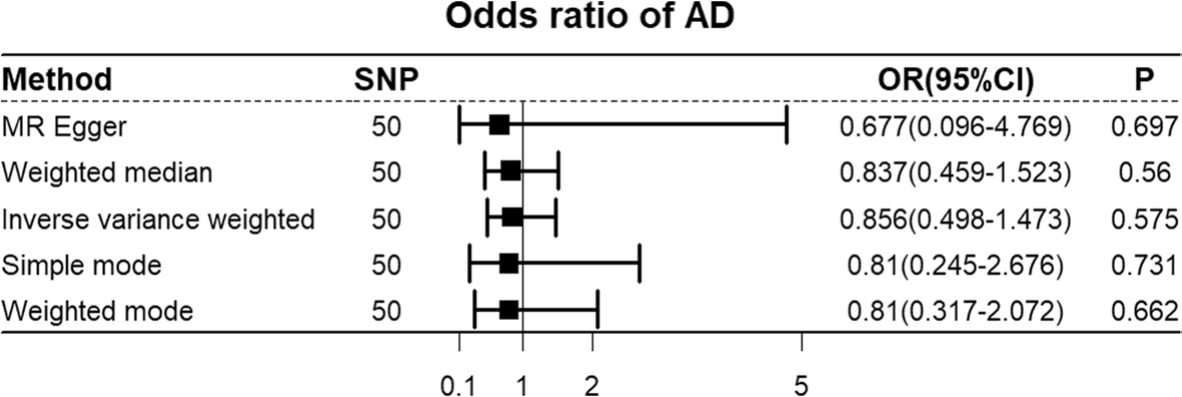
Summary of the Two Sample Mendelian Randomization-Based Analysis of Sleep Duration and Alzheimer's disease (AD)

We further investigated the Two Sample MR of sleep duration and AD. To remove IVs with linkage disequilibrium, SNPs were clumped for independence if they had a correlation of *r*^*2*^ > 0.001 [34]. Sensitivity analysis was performed using Two Sample MR R packages [35] and MR analysis using fixed/random effects weighted median, inverse variance weighted, weighted mode, simple mode, and MR-Egger regression. SNPs of sleep duration and AD were harmonized by *Harmonise_data ()* function from Two Sample MR R packages, and SNPs (rs1611719) for incompatible alleles and SNPs that were palindromic with intermediate allele frequencies (*rs*113021516, *rs*11643715, *rs*17732997, *rs*2079070, *rs*2186122, *rs*2279681, *rs*2683630, *rs*35662245, *rs*6561715, and *rs*72831782) were removed. A summary of the MR-based analysis of sleep duration and AD is shown in Fig. 5. Sensitivity analysis did not detect pleiotropy (MR-Egger intercept, *p* = 0.80) and the evidence of confounding heterogeneity of effect sizes (*p* < 0.05) was not noted in these two analyses. Therefore, random effects models were used to further verify the relationship. Inverse variance weighted-MR with random effect yielded similar causal association results (*p* > 0.05). Visualization of the Two Sample MR results is presented in Supplementary material Figure S2. A statistically causal association between sleep duration and AD was not observed in this study.

## Discussion

Based on the UKB, we conducted a large longitudinal study of 483,507 participants to study the association between sleep duration and AD risk. We had the following findings: 1) there was a there was a non-linear relationship between AD incidence and sleep duration (*P* for non-linear < 0.001) by restricted cubic splines (RCS) relationship between sleep duration and AD risk; 2) long sleep duration was significantly associated with a higher risk of AD, regardless of high, intermediate or low AD-GRS; 3) no causal association between AD and sleep duration using Two Sample MR.

Both genetic and lifestyle factors influence an individual's risk of developing AD and other subtypes of dementia [36]. In older adults without cognitive impairment or dementia, having a poor lifestyle and high GRS was significantly associated with a higher dementia risk, where GRS may be offset by certain lifestyle factors [37]. To our knowledge, this is the first study to investigate the effect of the interaction between sleep duration and GRS on AD events. Epidemiological studies have suggested there is a U- or J-shaped relationship between sleep duration and AD [8, 9, 38]. A non-linear relationship between sleep duration and AD risk was observed in our study. The risk of AD in the long sleep duration group was still higher than that of the normal sleep duration group after adjusting for multiple variables. Studies showed that people with AD had a longer sleep duration than older adults without cognitive impairment, and the association worsened as the disease progressed [39, 40]. In addition, a statistically causal association between sleep duration and AD was also not observed in our study by MR. Recent studies also showed that prolonged sleep duration was an early marker of neurodegeneration [41].

The mechanism of long sleep duration in dementia may be related to the changes in sleep and wakeup areas of the brain, including the suprachiasmatic nucleus between the pineal gland and the retina [42]. Levels of the wake-promoting neuropeptide hypocretin-1 have also been reported to be reduced in AD, as are the levels of hypocretin-1 neurons [43], which may be one of the mechanisms that cause prolonged sleep duration time in AD patients. In addition, because the preclinical phase of dementia lasts for at least 10 years [44], long sleep duration may be one of the clinical predictors of a higher risk of AD. Though there was no causal association between AD and sleep duration using Two Sample MR, long sleep duration was significantly associated with a higher risk of AD, regardless of high, intermediate or low AD-GRS.

Using Two Sample MR analysis method to further verify the causal relationship between sleep duration and AD was also one of the advantages of our study. In our observational study, while we corrected for major confounder factors including age, gender, ethnicity, TDI, education level, smoking, alcohol use, hypertension, stroke, myocardial infarction, and diabetes, residual confounding from unknown or unmeasured factors still remained possible. Since the observational study method had unknown or unmeasured confounding factors and could only show the correlation between sleep duration and AD, we further used Two Sample MR to avoid the effect of unknown or unmeasured confounding factors on sleep duration and AD correlation, and verify the causal relationship between sleep duration and AD. The direct acyclic graph (DAG) was shown in the supplementary material Figure S3.

This study has the following limitations. Data on patient sleep duration were first registered and there may be variations in the participants’ sleep duration not taken into account in this study. This study is UKB based, which has participants that are predominantly of European ancestry. This may affect the applicability of the results to other ethnicities, but does not change the internal validity of this study.

## Conclusions

In the UKB population, though there was no causal association between AD and sleep duration using Two Sample MR, long sleep duration was significantly associated with a higher risk of AD, regardless of high, intermediate or low AD-GRS. Prolonged sleep duration may be one of the clinical predictors of a higher risk of AD.

## Supplementary Information

**Additional file 1: Figure S1.** Study Sample Flow Diagram. **Figure S2.** Visualization of Mendelian randomization results. **Figure S3.** The direct acyclic graph (DAG) between sleep duration and AD.

**Additional file 2. **

## Data Availability

The data that support the findings of this study are available from UK Biobank but restrictions apply to the availability of these data, which were used under license for the current study, and so are not publicly available. Data are however available from the authors upon reasonable request and with permission of UK Biobank.

## Abbreviations

AD: Alzheimer's disease
UKB: UK Biobank
MR: Mendelian randomization
SNPs: Single nucleotide polymorphisms
GRS: Genetic risk score
IVs: Instrumental variables
GWASs: Genome-wide association studies
MR: Mendelian randomization
