# Supplementary material for: Sleep duration, genetic susceptibility, and Alzheimer's disease: a longitudinal UK Biobank-based study
Source: BMC Geriatr. 2022 Aug 2;22:638. doi: 10.1186/s12877-022-03298-8 (PMC9344659; doi:10.1186/s12877-022-03298-8)
Supplement: Supplementary file 1 — Additional file 1: Figure S1. Study Sample Flow Diagram. Figure S2. Visualization of Mendelian randomization results. Figure S3. The direct acyclic graph (DAG) between sleep duration and AD. [file 12877_2022_3298_MOESM1_ESM.docx]

**Figure S1. Study Sample Flow Diagram**


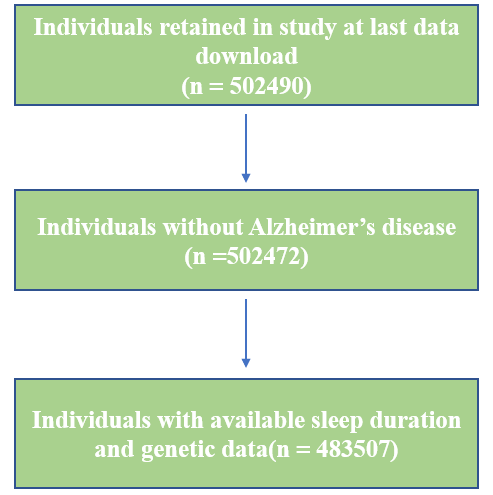


**Figure S2. Visualization of Mendelian randomization results**

1. scatter diagram;


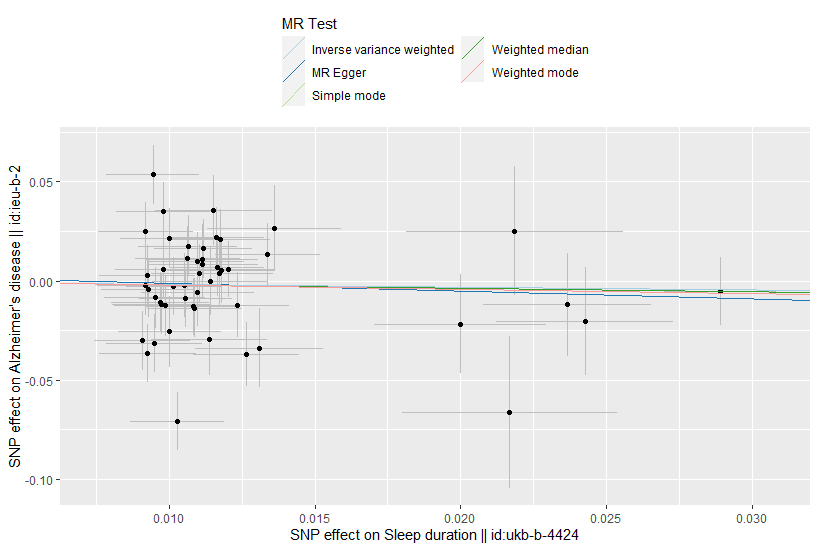


1. Forest map


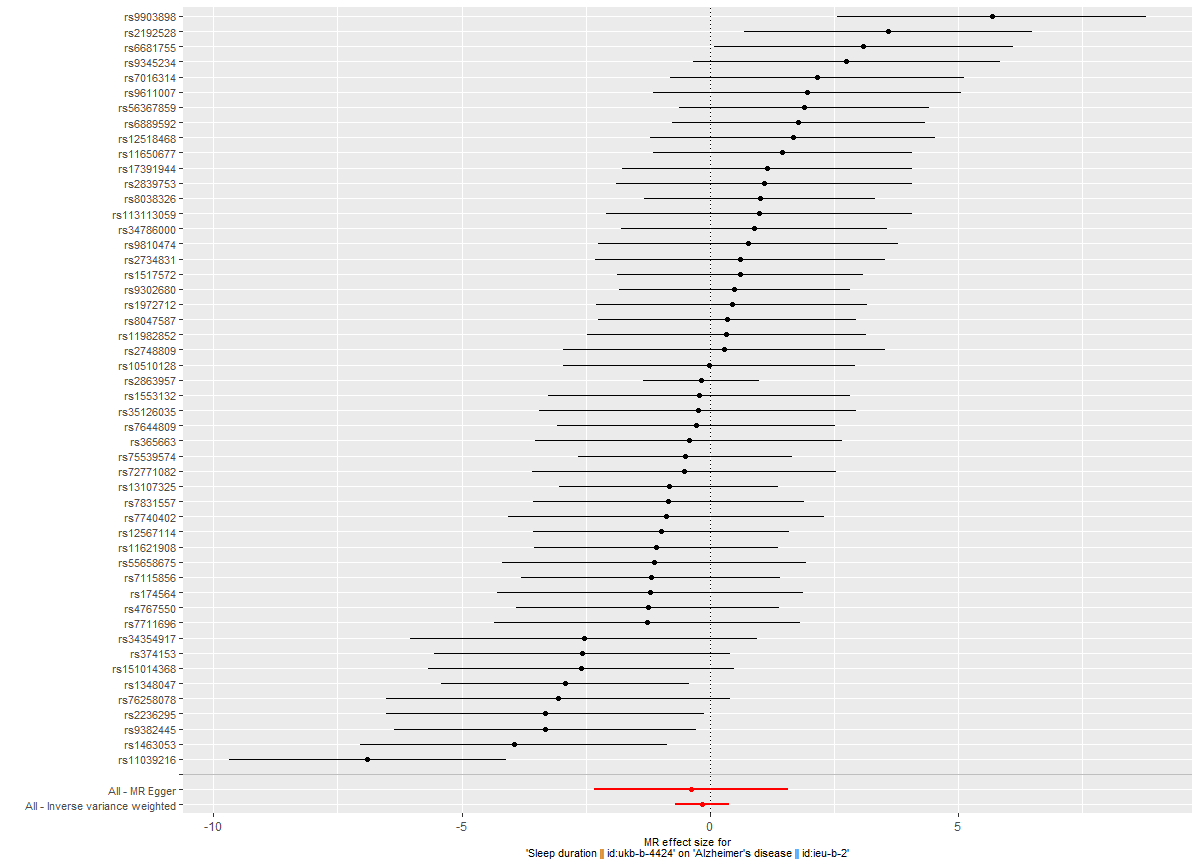


1. Draw sensitivity analysis diagram


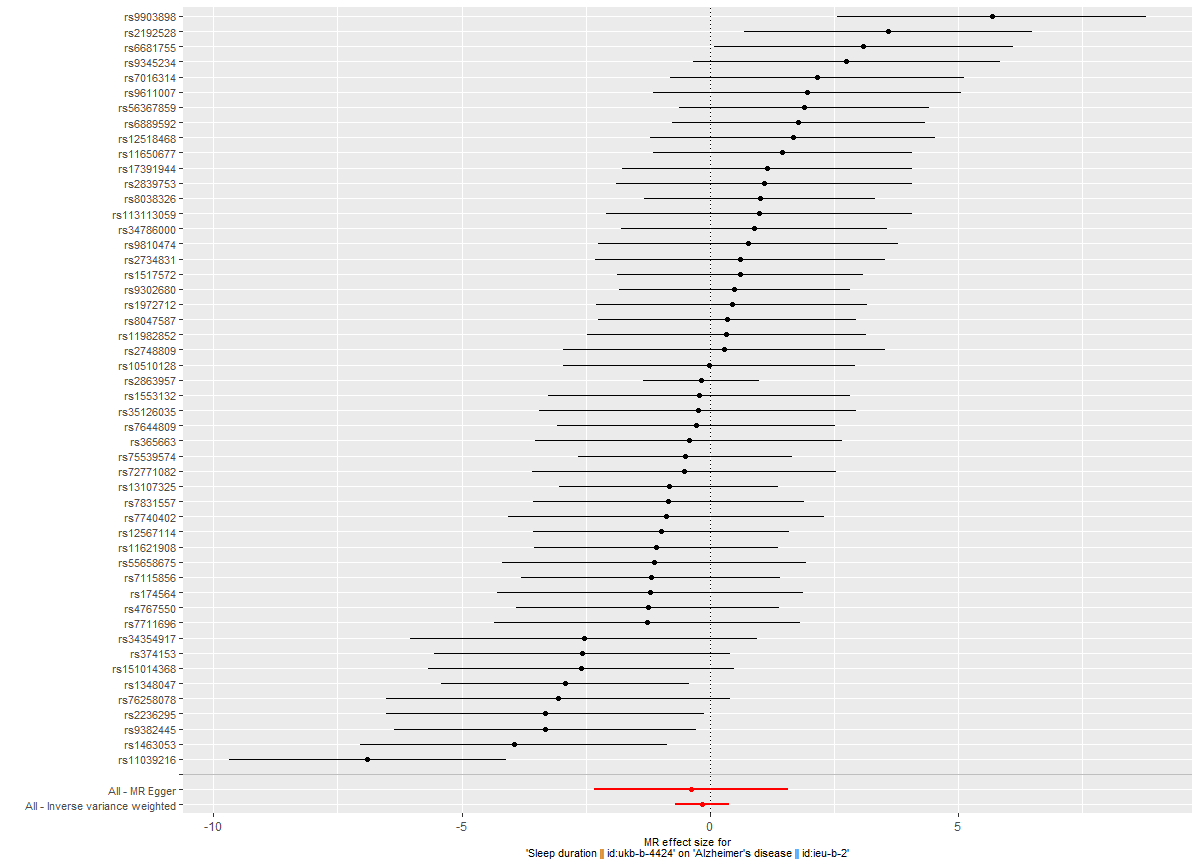


(4) Funnel plot


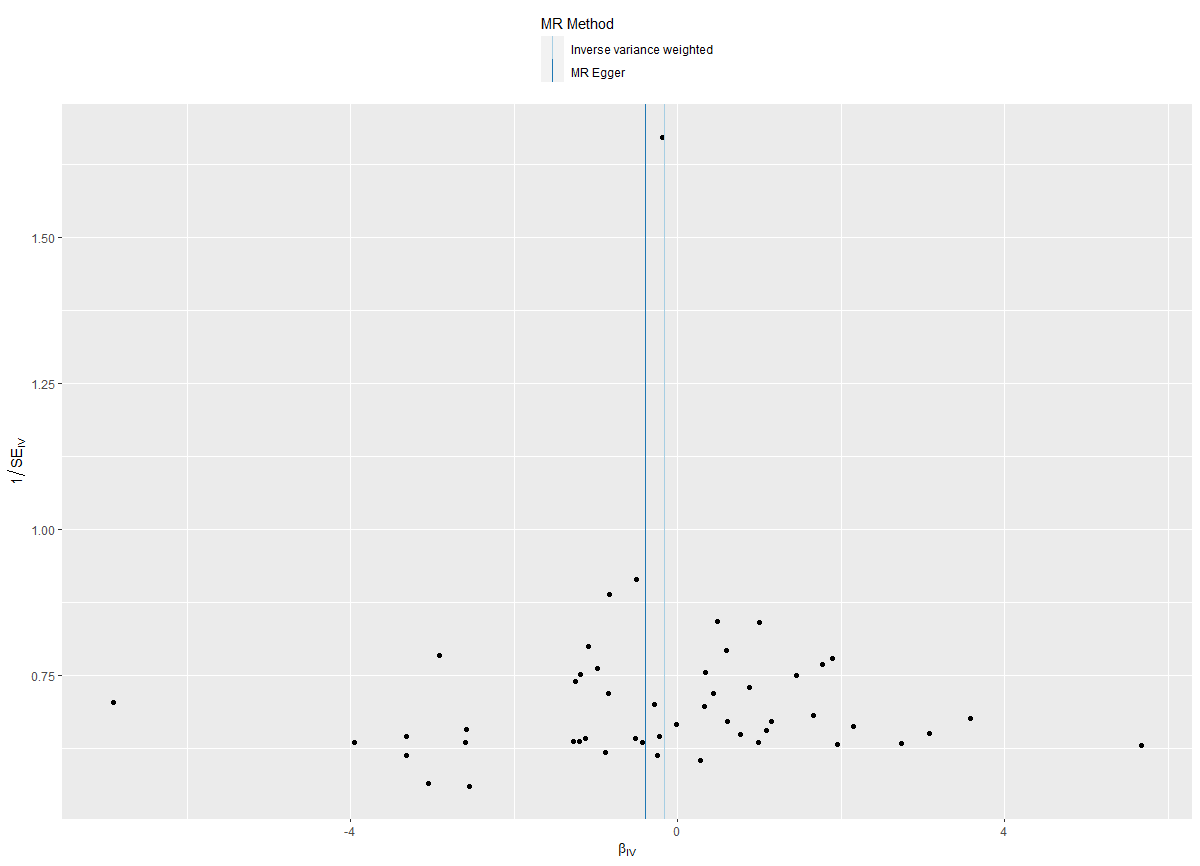


**Figure S3. The direct acyclic graph (DAG) between sleep duration and AD**


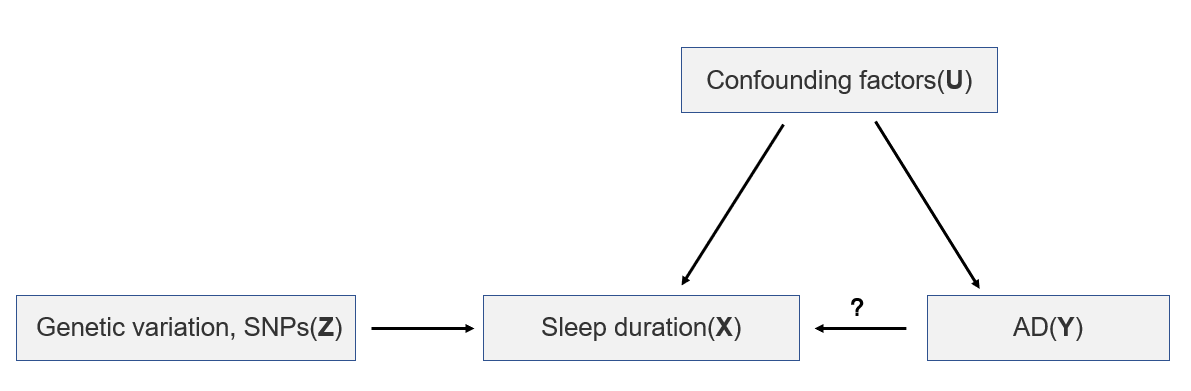


**Z:** SNPs of sleep duration

**X:** Expose (sleep duration)

**U**: Confounding factors

**Y:** Outcome (AD)

**?:** Possible paths
